# Carpenter on Alcoholic Liquors

**Published:** 1854-02

**Authors:** 


*Carpenter on Alcoholic Liquors.*

This excellent essay of Dr. Carpenter has been published by Messrs. Blanchard & Lea, Philadelphia, in a convenient form for mailing. Those interested in the subject of temperance, will find it replete with physiological facts, expressed in language easily understood by all.

To societies and others, desirous of distributing the work, the publishers propose to furnish it at reduced prices.

For sale by Cooke & Co. J.

